# List of the names of the referees for the studies that were published in the São Paulo Medical Journal/Evidence for Health Care during the year 2011

**DOI:** 10.1590/S1516-31802011000600014

**Published:** 2011-12-01

**Adolfo Wenjaw Liao:** Faculdade de Medicina da Universidade de São Paulo (FMUSP)

**Alfredo Carlos Barros:** Hospital Israelita Albert Einstein (HIAE)

**Ana Beatriz de Azevedo:** Universidade Federal de São Paulo (Unifesp)

**Andreza Feitosa Ribeiro:** Hospital Israelita Albert Einstein (HIAE)

**Angela Francisca Trinconi:** Instituto do Câncer do Estado de São Paulo (ICESP)

**Antonio José Gonçalves:** Faculdade de Ciências Médicas da Santa Casa de São Paulo (FCMSCSP)

**Auro del Giglio:** Faculdade de Medicina da Fundação ABC

**Aytan Miranda Sipahi:** Hospital das Clínicas da Faculdade de Medicina da Universidade de São Paulo (HCFMUSP)

**Benoit Jacques Bibas:** Hospital das Clínicas da Faculdade de Medicina da Universidade de São Paulo (HCFMUSP)

**Carlos Alberto Ruiz:** Hospital das Clínicas da Faculdade de Medicina da Universidade de São Paulo (HCFMUSP)

**Carlos Eduardo Martinelli Junior:** Faculdade de Medicina de Ribeirão Preto da Universidade de São Paulo (FMRP/USP)

**Carolina Araújo Rodrigues Funayama:** Faculdade de Medicina de Ribeirão Preto da Universidade de São Paulo (FMRP/USP)

**Cristina Muccioli:** Universidade Federal de São Paulo (Unifesp)

**Celso Gromatzky:** Faculdade de Medicina do ABC (FMABC)

**Delcio Matos:** Universidade Federal de São Paulo (Unifesp)

**Domingo Marcolino Braile:** Faculdade de Medicina de São José do Rio Preto (Famerp) and Faculdade de Ciências Médicas da Universidade Estadual de Campinas (FCM-Unicamp)

**Ediléia Bagatin:** Universidade Federal de São Paulo (Unifesp)

**Edina Mariko Koga da Silva:** Universidade Federal de São Paulo (Unifesp)

**Eduardo Maia Freese de Carvalho:** Centro de Pesquisas Aggeu Magalhães, Fundação Oswaldo Cruz (CPqAM/Fiocruz)

**Eduardo Vieira Motta:** Hospital das Clínicas da Faculdade de Medicina da Universidade de São Paulo (HCFMUSP)

**Emil Burihan:** Universidade Federal de São Paulo (Unifesp)

**Edward Araújo Júnior:** Universidade Federal de São Paulo (Unifesp)

**Elcio Oliveira Vianna:** Faculdade de Medicina de Ribeirão Preto da Universidade de São Paulo (FMRP/USP)

**Emmanuel de Almeida Burdmann:** Faculdade de Medicina da Universidade de São Paulo (FMUSP)

**Fernando Antonio de Almeida:** Faculdade de Ciências Médicas e da Saúde (FCMS), Pontifícia Universidade Católica de São Paulo (PUC-SP)

**Fernando Ferreira Costa:** Faculdade de Ciências Médicas da Universidade Estadual de Campinas (FCM-Unicamp)

**Flávio Faloppa:** Universidade Federal de São Paulo (Unifesp)

**Francisco Estivallet Finamor Júnior:** Universidade Federal de São Paulo (Unifesp)

**Gianni Mara Silva dos Santos:** Universidade Federal de São Paulo (Unifesp)

**Hernani Pinto de Lemos Júnior:** Universidade Federal de São Paulo (Unifesp)

**Irineu Tadeu Velasco:** Faculdade de Medicina da Universidade de São Paulo (FMUSP)

**Jamil Natour:** Universidade Federal de São Paulo (Unifesp)

**João Carlos Campos Guerra:** Hospital Israelita Albert Einstein (HIAE) and Centro de Hematologia de São Paulo (CHSP)

**João Renato Rebello Pinho:** Faculdade de Medicina da Universidade de São Paulo (FMUSP)

**José Antônio Rocha Gontijo:** Faculdade de Ciências Médicas da Universidade Estadual de Campinas (FCM-Unicamp)

**José Carlos Nicolau:** Faculdade de Medicina da Universidade de São Paulo (FMUSP)

**José Geraldo Mill:** Universidade Federal do Espírito Santo (UFES)

**José Luiz Barbosa Bevilacqua:** Hospital Sírio-Libanês (HSL)

**José Roberto Filassi:** Faculdade de Medicina da Universidade de São Paulo (FMUSP) and Instituto do Câncer do Estado de São Paulo (ICESP)

**José Roberto Lapa e Silva:** Universidade Federal do Rio de Janeiro (UFRJ)

**Júlio César Rodrigues Pereira:** Faculdade de Saúde Pública (FSP), Universidade de São Paulo (USP)

**Kodi Kojima:** Hospital das Clínicas da Faculdade de Medicina da Universidade de São Paulo (HCFMUSP)

**Laércio Joel Franco:** Faculdade de Medicina de Ribeirão Preto da Universidade de São Paulo (FMRP/USP)

**Leonardo Rolim Ferraz:** Hospital Israelita Albert Einstein (HIAE)

**Lorena Lobo de Figueiredo-Pontes:** Faculdade de Medicina de Ribeirão Preto da Universidade de São Paulo (FMRP/USP)

**Luiz Antonio Nogueira-Martins:** Universidade Federal de São Paulo (Unifesp)

**Luiz Henrique Martins Castro:** Faculdade de Medicina da Universidade de São Paulo (FMUSP)

**Luiz Jacintho Da Silva:** International Vaccine Institute

**Lydia Masako Ferreira:** Universidade Federal de São Paulo (Unifesp)

**Marcelo Annes:** Universidade Federal de São Paulo (Unifesp)

**Marco Aurélio Santo:** Hospital das Clínicas da Faculdade de Medicina da Universidade de São Paulo (HCFMUSP)

**Maria Cristina Pereira Lima:** Faculdade de Medicina de Botucatu da Universidade Estadual Paulista (FMB-Unesp)

**Maria José Carvalho Carmona:** Faculdade de Medicina da Universidade de São Paulo (FMUSP)

**Maria Regina Torloni:** Universidade Federal de São Paulo (Unifesp)

**Marilza Vieira Cunha Rudge:** Faculdade de Medicina de Botucatu da Universidade Estadual Paulista (FMB-Unesp)

**Mario Augusto Taricco:** Faculdade de Medicina da Universidade de São Paulo (FMUSP)

**Mario Ferreira Junior:** Hospital das Clínicas da Faculdade de Medicina da Universidade de São Paulo (HCFMUSP)

**Milton de Arruda Martins:** Faculdade de Medicina da Universidade de São Paulo (FMUSP)

**Moacir Fernandes de Godoy:** Faculdade de Medicina de São José do Rio Preto (FAMERP)

**Morgani Rodrigues:** Hospital Israelita Albert Einstein (HIAE)

**Nelson Adami Andreollo:** Faculdade de Ciências Médicas da Universidade Estadual de Campinas (FCM-Unicamp)

**Noedir Antonio Groppo Stolf:** Faculdade de Medicina da Universidade de São Paulo (FMUSP)

**Olavo Pires de Camargo:** Faculdade de Medicina da Universidade de São Paulo (FMUSP)

**Onivaldo Cervantes:** Escola Paulista de Medicina - Universidade Federal de São Paulo (EPM-Unifesp)

**Orsine Valente:** Universidade Federal de São Paulo (Unifesp)

**Patrícia Coelho de Soárez:** Faculdade de Medicina da Universidade de São Paulo (FMUSP)

**Paulo Cezar Feldner Júnior:** Universidade Federal de São Paulo (Unifesp)

**Paulo Herman:** Hospital das Clínicas da Faculdade de Medicina da Universidade de São Paulo (HCFMUSP)

**Paulo Manuel Pêgo Fernandes:** Hospital das Clínicas da Faculdade de Medicina da Universidade de São Paulo (HCFMUSP)

**Rachel Riera:** Universidade Federal de São Paulo (Unifesp)

**Raul Cutait:** Faculdade de Medicina da Universidade de São Paulo (FMUSP)

**Regina Carvalho Pinto:** Faculdade de Medicina do ABC

**Regina Célia Mello Santiago Moisés:** Universidade Federal de São Paulo (Unifesp)

**Ricardo Baladi Rufino Pereira:** Universidade Federal de São Paulo (Unifesp)

**Ricardo Mingarini Terra:** Hospital das Clínicas da Faculdade de Medicina da Universidade de São Paulo (HCFMUSP)

**Roberto Alexandre Franken:** Faculdade de Ciências Médicas da Santa Casa de São Paulo (FCMSCSP)

**Rosa Aparecida Ferreira:** Centro Universitário Barão de Mauá

**Ruy Laurenti:** Faculdade de Medicina da Universidade de São Paulo (FMUSP)

**Ruth Caldeira Melo:** Escola de Artes, Ciências e Humanidades, Universidade de São Paulo (EACH/USP)

**Sérgio Tufik:** Escola Paulista de Medicina — Universidade Federal de São Paulo

**Soubhi Kahhale:** Faculdade de Medicina da Universidade de São Paulo (FMUSP)

**Stella Regina Taquette:** Universidade Estadual do Rio de Janeiro (UERJ)

**Sumaia Inaty Smaira:** Faculdade de Medicina de Botucatu da Universidade Estadual Paulista (FMB-Unesp)

**Túlio Diniz Fernandes:** Faculdade de Medicina da Universidade de São Paulo (FMUSP)

**Vanessa Pedrosa Vieira:** Universidade Federal de São Paulo (Unifesp)

**Vicente Odone Filho:** Faculdade de Medicina da Universidade de São Paulo (FMUSP)

**Virgínia Paes Leme Ferriani:** Faculdade de Medicina de Ribeirão Preto da Universidade de São Paulo (FMRP/USP)

**Walter José Gomes:** Universidade Federal de São Paulo (Unifesp)

**Wanderley Marques Bernardo:** Faculdade de Medicina da Universidade de São Paulo (FMUSP)

